# Prognostic significance of HDL-C on long-term mortality in patients with COVID-19 pneumonia in the Turkish population: A potential mechanism for population differences

**DOI:** 10.17305/bjbms.2022.7545

**Published:** 2022-08-22

**Authors:** Ömer Faruk Baycan, Furkan Bölen, Başak Atalay, Mehmet Agirbasli

**Affiliations:** 1 Department of Cardiology, Istanbul Medeniyet University, Göztepe Prof. Dr. Suleyman Yalcin City Hospital, Istanbul, Turkey; 2 Department of Radiology, Istanbul Medeniyet University Göztepe Prof. Dr. Suleyman Yalcin City Hospital, Istanbul, Turkey

**Keywords:** COVID-19, HDL-C, lipoproteins, biomarkers, personalized medicine, public health

## Abstract

Coronavirus disease 2019 (COVID-19) is diagnosed by the evidence of the presence of multiple phenotypes, including thrombosis, inflammation, and alveolar and myocardial damage, which can cause severe illness and mortality. High-density lipoprotein cholesterol (HDL-C) has pleiotropic properties, including anti-inflammatory, anti-infectious, antithrombotic, and endothelial cell protective effects. The aim of this study was to investigate the HDL-C levels and one-year mortality after the first wave of patients with COVID-19 was hospitalized. Data from 101 patients with COVID-19 were collected for this single-center retrospective study. Lipid parameters were collected on the admission. The relationship between lipid parameters and long-term mortality was investigated. The mean age of the non-survivor group (n = 38) was 68.8 ± 14.1 years, and 55% were male. The HDL-C levels were significantly lower in the non-survivors group compared with the survivors (26.9 ± 9.5 vs 36.8 ± 12.8 mg/dl, respectively *p* < 0.001). Multivariate regression analysis determined that age, C-reactive protein, D-dimer, hypertension, and HDL-C as independent predictors for the development of COVID-19 mortality. HDL-C levels <30.5 mg/dl had 71% sensitivity and 68% specificity to predict one-year mortality after COVID-19. The findings of this study showed that HDL-C is a predictor of one-year mortality in Turkish patients with COVID-19. COVID-19 is associated with decreased lipid levels, and it is an indicator of the inflammatory burden and increased mortality rate. The consequences of long-term metabolic dysregulations in patients that have recovered from COVID-19 still need to be understood.

## INTRODUCTION

Coronavirus disease 2019 (COVID-19) is caused by severe acute respiratory syndrome coronavirus 2 (SARS-CoV-2) [1]. COVID-19 is diagnosed by the evidence of multiple phenotypes, including thrombosis, inflammation, and alveolar and myocardial damage, which can cause severe illness and mortality [2]. The disease course of COVID-19 is highly heterogeneous. Patients can present as completely asymptomatic or as having a mild influenza-like illness. In some cases, COVID-19 can progress rapidly into an extensive lung injury that requires intubation [3]. Over the past two years, several developments have modulated the disease phenotype of COVID-19. The availability of effective vaccines and resources, effective treatment algorithms, and the identification of novel variants with different clinical features have all added to the complexity of the COVID-19 phenotype. The SARS-CoV-2 disease targets the endothelium [4]. The expressions of serine proteases in human cells and angiotensin-converting enzyme 2 provide pathogenic mechanisms for SARS-CoV-2 cellular entrance and replication [5,6]. Comorbidities, such as hypertension (HTN) and diabetes mellitus (DM), are associated with a poor prognosis in COVID-19 [7-9].

Host defense mechanisms against viral infection and disease progression in COVID-19 remain to be elucidated. High-density lipoprotein cholesterol (HDL-C) has pleiotropic effects in infectious diseases [10,11]. The current understanding of the role of HDL-C in COVID-19 is slowly unfolding [10]. HDL-C plays a critical role in the immune system due to its pleiotropic effects [11]. HDL-C sequesters pathogen lipids, such as lipopolysaccharide, which mediate the dysregulated inflammation and organ dysfunction in sepsis [12]. Therefore, differences in HDL-C levels and function can potentially contribute to individual variabilities in the mortality of COVID-19. HDL-C has both antioxidant and anti-inflammatory effects [13]. Recombinant HDL-C therapy has become a new treatment for severe sepsis [14]. Patients with severe COVID-19 and mortality have been displayed to have lower HDL-C concentrations [15]. As society enters a post-COVID-19 era, the determinants of short- and long-term mortality from the first wave of COVID-19 are of interest.

Three decades ago, the Turkish Heart Study reported that the Turkish population had very low levels of HDL-C. The low HDL-C levels in Turkey were caused, at least in part, by genetic factors [16]. While research has been heavily focused on viruses and vaccines for the past two years, it is now understood that the host’s immune responses are just as important in determining the outcomes of COVID-19.

Protective proteins, such as HDL, are crucial in understanding all aspects of host defense. The effect of HDL-C in COVID-19 has been investigated previously [11,17,18], but its long-term prognostic significance has not yet been investigated. Populational differences exist in HDL-C levels and COVID-19 outcomes. The studies of the association of HDL-C levels and COVID-19 outcomes remain of clinical interest.

This study aims to investigate the effect of HDL-C on long-term prognosis in patients with COVID-19.

## MATERIALS AND METHODS

### Study population

**FIGURE 1:**
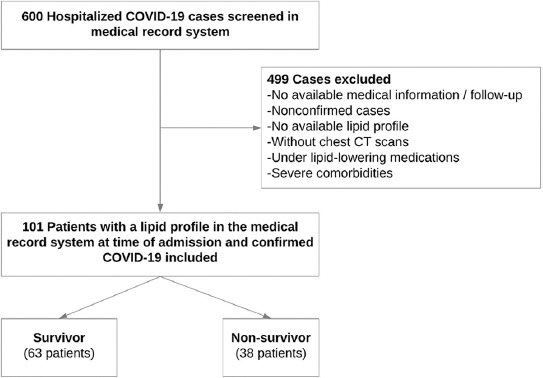
Flowchart of the study.

This retrospective, single-center, cohort study included 101 patients hospitalized for COVID-19 pneumonia between 1 April 2020 and 31 December 2020. There was a significant surge in the number of cases in Turkey during the first wave of the COVID-19 outbreak. The vaccination process in Turkey started early in 2021 with the CoronaVac vaccine. The Pfizer-BioNTech vaccine doses were available later in 2021. None of the patients was vaccinated at the time of the study or had recurrent disease. None of the patients had the time to complete full dose vaccination by the time of follow-up. Patients with missing information (i.e., their lipid levels or a chest-computerized tomography [CT] during the same admission) or who had a negative polymerase chain reaction (PCR) test result for SARS-CoV-2 were excluded from the study. Patients with chronic kidney disease (glomerular filtration rate <30 ml/min), chronic obstructive lung disease, an interstitial lung disease with reduced total lung capacity, history of cancer, underlying terminal illness, and use of lipid-lowering medications were excluded from the study. The study population is shown as a flow chart in Figure 1.

The following criteria were used for inclusion in the study: Age >18; positive PCR test for SARS-CoV-2; diagnosis of COVID-19 pneumonia; laboratory tests that included lipid levels on hospital admission. We only included patients who completed chest X-ray and chest CT in the same hospital admission. Patient data were obtained from the hospital medical record system. The correlation between the lipid parameters and disease-related indices was analyzed.

### PCR test for COVID-19 diagnosis

Reverse transcription PCR (RT-PCR) test kits provided by the Ministry of Health, using Biospeedy COVID-19 RT-qPCR, were used to establish a diagnosis of COVID-19. Swab samples, including viral transport mediums, were studied using the RT-qPCR method according to the manufacturer’s instructions (Bioeksen, Istanbul, Turkey). The test kit detects the SARS-CoV-2 RNA-dependent RNA polymerase (RdRp) single-gene fragment, which is specific to SARS-CoV-2 (FAM Channel). On 19 March 2020, the World Health Organization published a document that declared the “single discriminatory target is considered sufficient in areas where COVID-19 virus is widely spread.” The human Ribonuclease P was selected as the internal control (HEX channel). Gene amplification reactions and signal detections were performed in the LightCycler 480 System (Roche Diagnostics GmbH, Mannheim, Germany). Samples with a positive signal in the internal control and RdRp region were considered positive, while samples with a positive signal in the internal control and a negative result in the RdRp region were considered negative. Cycle threshold (Ct) values that were below 40 were considered positive. If the Ct value was zero, the test was considered negative. The PCR tests were conducted approximately 16 h after the test was ordered.

### Grading of computerized tomography evidence of disease

A chest CT severity score (CT-SS) was developed to assess the severity of COVID-19 pneumonia in all cases. Yang et al. described the staging of the CT-SS [19]. The CT-SS includes ground glass appearances, crazy paving patterns, and consolidations. The 18 segments of both lungs were divided into 20 regions. The lung opacities in all 20 lung regions were subjectively evaluated for the chest CT using a system that attributed scores of 0, 1, or 2, depending on a parenchymal opacification of 0%, <50%, or >50%, respectively. The CT-SS is calculated as the sum of the scores in the 20 lung segments, ranging from 0 to 40 [19]. This study used a combination of clinical assessments, thorax CTs, and RT-PCRs to diagnose COVID-19 [20].

### Biochemical examinations

The lipid profile and glucose level were measured using the enzymatic colorimetric-assay method in an automated analyzer (Abbott Architect c16000). An automated hematology analyzer was used for the complete blood count analysis. The participants were asked to fast for 12 hours prior to the blood draw. The blood samples were stored at 4°C before the analysis that took place within 12 hours of retrieval. A low HDL-C level was defined based on the international criteria (<40 mg/dl or <50 mg/dl in men and women, respectively). Non-HDL-C levels were calculated as the total cholesterol (TC) level minus the HDL-C level. The cutoff value for the triglycerides (TG) was defined as 150 mg/dl [21].

### HDL-C assay

The fasting blood was drawn and stored at –80°C for analysis. The HDL-C values were measured using Roche Cobas 6000 automated platform with a c501 analyzer using Food and Drug Administration-approved HDL-C kits from Roche Diagnostics, GmbH, Mannheim, Germany. The Roche HDLC4 test principle is homogeneous enzymatic colorimetric and also a direct method. In this assay, non-HDL lipoproteins, such as low-density lipoprotein (LDL), very low-density lipoprotein (VLDL), and chylomicrons are combined with polyanions and a detergent forming a water-soluble complex. The HDL-C reacts with cholesterol esterase, cholesterol oxidase, and peroxidase to form a colored pigment that is measured optically.

### Follow-up and endpoints

The primary endpoint was the mortality rate at the end of year one. Patient data and mortality information were obtained from the hospital medical records and health registry system. The associations between HDL-C levels and disease severity indices such as CT-SS and inflammatory biomarkers were studied as secondary endpoints in the study.

### Ethical statement

The study was approved by the authors’ institutional research ethics committee and the Turkish Ministry of Health (protocol number 2020-06-02T18_44_32) in accordance with the institutional and national research ethics standards. The study was conducted in accordance with the principles expressed in the Declaration of Helsinki. The Institutional Review Board approved the study protocol.

### Statistical analysis

The Kolmogorov–Smirnov test was used to analyze the normality of the data. Continuous variables with a normal distribution were expressed as the mean ± standard deviation, and variables with a nonparametric distribution were expressed as the median (interquartile range). Categorical variables were expressed as percentages when appropriate. A non-parametric Mann–Whitney U test was used to compare the unpaired samples with the nonparametric distribution, and Student’s t-test was used to compare the parametric with a normal distribution. After performing the univariate analysis, variables with a statistical significance were selected for the multivariate logistic regression analysis using the stepwise method. The results of the univariate and multivariate regression analyses were presented as the odds ratio (OR) with a 95% confidence interval (CI). *p* < 0.05 was considered statistically significant. Power analysis was performed to calculate the sample size by taking into account the previous studies in the literature. The minimum acceptable probability of preventing type I and II errors was 95% and 80%, respectively. Assuming the normal HDL-C level of 50 mg/dl and a 25% difference between the groups, the minimum sample size is calculated as 76. The statistical analysis was performed using the SPSS program (version 20.0 for Windows, SPSS Inc. Chicago, IL).

## RESULTS

### The study population

The clinical and demographic characteristics of 101 hospitalized COVID-19 pneumonia patients included in the study are presented in Table 1. The mean population age was 62.5 ± 16.5 years (56/45, Male/Female). Comorbidities were commonly observed in the study group, including HTN (45%), DM (33%), and coronary artery disease (19%). Patients were divided into two groups according to mortality at year one. Mortality was observed in 38 (37.6%) patients at the one-year stage. Laboratory findings, including hemoglobin (Hgb), lymphocyte, neutrophil to lymphocyte ratio (NLR), creatinine, urea, C-reactive protein (CRP), procalcitonin, high-sensitivity troponin I (hs-TnI), and D-dimer levels, were significantly different between the non-survivor and the survivor groups (*p* < 0.05). HTN was observed significantly more frequently in patients in the non-survivor group than in the survivors (*p* = 0.025). There was no statistically significant difference in CT-SS between the groups. The patients were compared according to the treatments received during COVID-19. The use of steroids and angiotensin-converting-enzyme inhibitors/angiotensin-receptor blockers was higher in the non-survivor group than in the survivor group (Table 1).

**TABLE 1:**
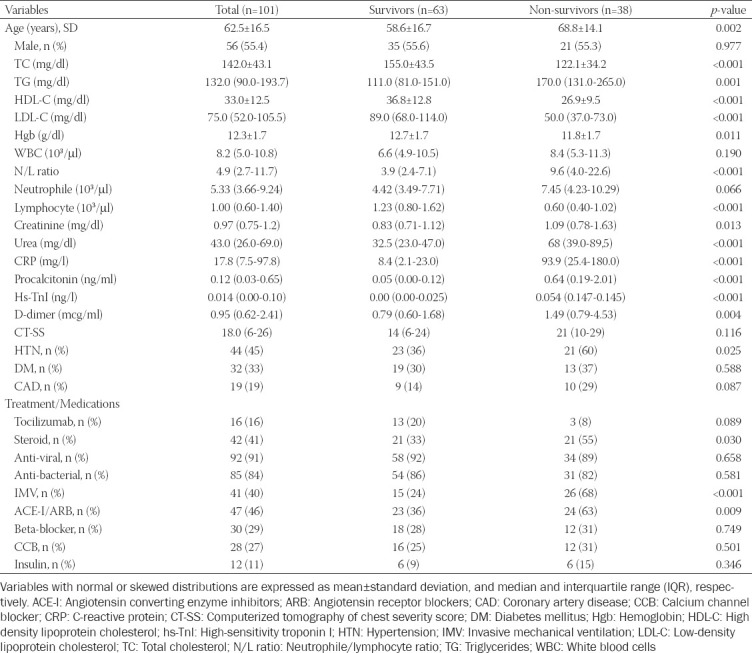
Demographic and clinical characteristics of survivor/non-survivor patients

### Lipid levels

**FIGURE 2:**
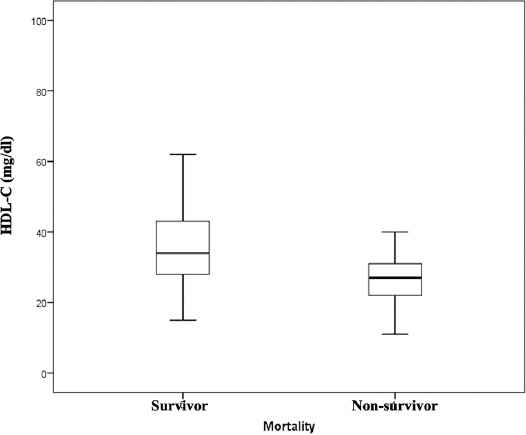
Box plot figure displays that HDL-C levels are significantly lower in non-survivors compared to the survivors (26.9 ± 9.5 vs 36.8 ± 12.8, *p* < 0.001, respectively). HDL-C: High-density lipoprotein cholesterol.

The patients included in the study were compared according to their lipid levels. The TC, LDL-C, and HDL-C levels were lower (*p* < 0.001) in the non-survivor group than in the survivor group, while TG levels were higher (*p* = 0.001) (Table 1 and Figure 2).

### Predictors of mortality 

A logistic regression analysis was performed to find the predictors of mortality. The covariates in univariate analyses were the patients’ age, gender, HDL-C, TC, DM, HTN, CAD, hs-TnI, procalcitonin, CRP, Hgb, CT-SS, creatinine, urea, NLR, D-dimer, and steroid use. Parameters that were significant in univariate analysis were evaluated with multivariate analysis. Age (OR: 1.056, 95% Cl: 1.182-1.323; *p* = 0.004), HDL-C (OR: 0.90, 95% Cl: 0.852-0.957; *p* = 0.023), HTN (OR: 2.985, 95%Cl: 1.048-8.025; *p* = 0.041), CRP (OR: 1.018, 95% Cl: 1.001-1.035; *p* = 0.003), and D-dimer (OR: 1.112, 95% Cl: 1.001-1263; *p* = 0.039) were significant parameters that were associated with long-term mortality (Table 2). The measure of the predictive power of this regression model according to Nagelkerke R-square is good (69%) (R^2^_N_: 0.692), and also the goodness of fit of the Theron model according to the Chi-square test is present (Chi-square: 56.1; degrees of freedom: 9; *p* < 0.001). These results showed that the regression model used for this study was correctly specified.

**TABLE 2:**
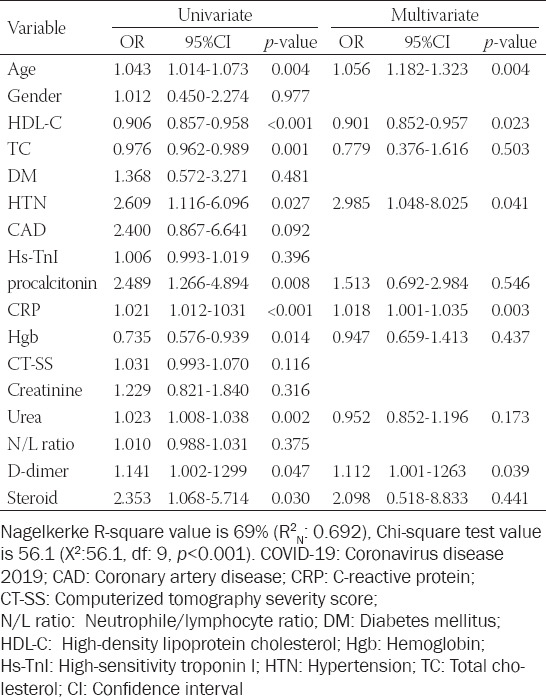
Univariate and multivariate analyses of predictors of mortality in patients with COVID-19 pneumonia

**FIGURE 3:**
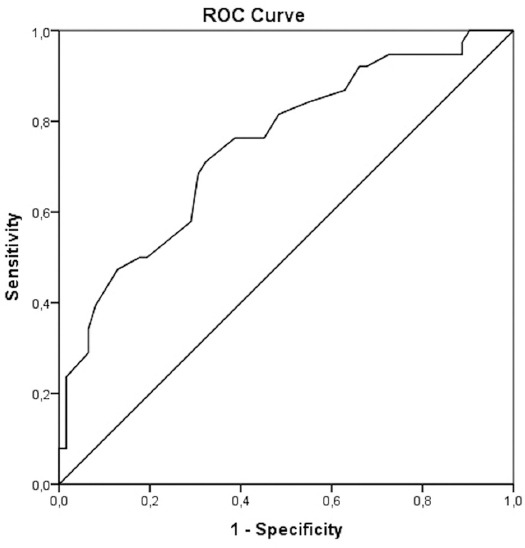
The specificity and sensitivity of the cutoff values for HDL-C are evaluated by ROC analysis to predict the long-term mortality. The AUC was measured as 0.75 (95% CI 0.65-0.84, *p* = 0.001) for HDL-C. HDL-C had 71% sensitivity and 68% specificity to predict the mortality at year one with a cutoff value of 30.5 mg/dl. ROC: Receiver operating characteristics; HDL-C: High-density lipoprotein cholesterol; AUC: Area under the curve.

**FIGURE S1:**
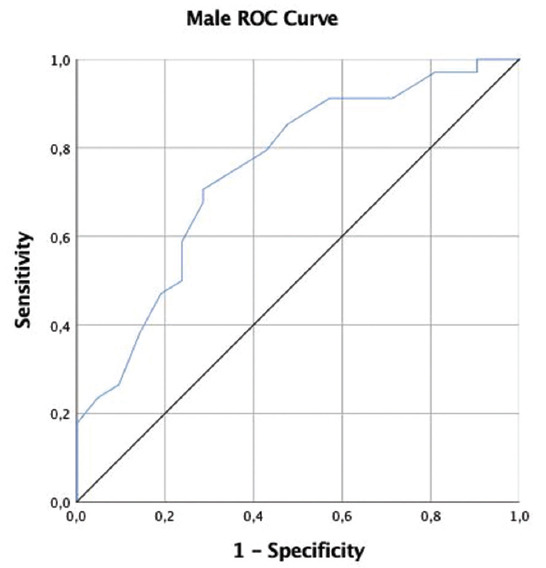
The specificity and sensitivity of the cutoff values for HDL-C are evaluated by ROC analysis to predict the long-term mortality for male patients. The best performing value of HDL-C (29.5 mg/ dL) in male patients to predict one-year mortality was associated with 70% sensitivity and 72% specificity. The AUC of the HDL-C for predicting mortality was 0.749 (95% CI 0.614–0.883; p = 0.002). HDL-C: high-density lipoprotein cholesterol; AUC: area under the curve

**FIGURE S2:**
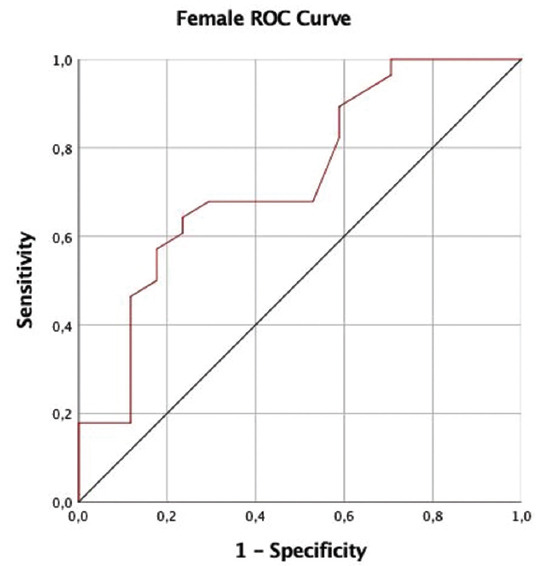
The specificity and sensitivity of the cutoff values for HDL-C are evaluated by ROC analysis to predict the long-term mortality for female patients. The best performing value of HDL-C (30.5 mg/dL) in female patients in predicting one-year mortality was associated with 68% sensitivity and 71% specificity. The AUC of the HDL-C for predicting mortality was 0.730 (95% CI 0.576–0.884; p = 0.01). HDL-C: high-density lipoprotein cholesterol; AUC: area under the curve

The specificity and sensitivity of the cutoff values for HDL-C were evaluated using the receiver operating characteristic analysis to predict mortality at year one. The areas under the curve (AUC) were measured as 0.75 for HDL-C (95%CI: 0.65–0.84, *p* = 0.001). The HDL-C had 71% sensitivity and 68% specificity in predicting mortality at year one, with a cutoff value of 30.5 (mg/dl) (Figure 3). The HDL-C levels were lower in men than in women (30.56 ± 9.27 mg/dl vs 35.93 ± 15.16 mg/dl; *p* = 0.041) (Table S1). The best performing value of HDL-C (29.5 mg/dl) in male patients to predict mortality was associated with 70% sensitivity and 72% specificity. The AUC of the HDL-C for predicting mortality was 0.749 (95%CI: 0.614–0.883; *p* = 0.002) (Figure S1). The best performing value of HDL-C (30.5 mg/dl) in women patients in predicting mortality was associated with 68% sensitivity and 71% specificity. The AUC of the HDL-C for predicting mortality was 0.730 (95%CI: 0.576–0.884; *p* = 0.01) (Figure S2).

## DISCUSSION

The effects of HDL-C on the COVID-19 outcome are of interest. In this study, we aimed to show the effect of HDL-C in patients with COVID-19 on their long-term prognosis. The main findings of our study are that, first, TC, LDL-C, and HDL-C were lower, and TG levels were higher in the ­non-survivor group compared with the survivors. Second, age, CRP, D-dimer, HTN, and HDL-C were independent predictors of mortality. Third, HDL-C levels below 30.5 mg/dl had a 71% sensitivity and 68% specificity to predict one-year mortality after COVID-19.

The previous studies report that age is a major risk factor for COVID-19 and adverse health outcomes, including hospitalization, intensive care unit admission, and death [22]. Similarly, CRP and D-dimer levels are associated with the disease progression and severity of COVID-19 and may predict mortality in hospitalized patients [23,24]. Recent meta-analyzes to identify at-risk populations with poor prognosis found that age, male sex, DM, and HTN were associated with higher mortality [7,25]. This study confirms the findings of the previous studies by identifying age, CRP, D-dimer, and HTN as predictors of one-year mortality. Conflicting reports exist on the association between HDL-C levels and poor COVID-19 outcomes [26]. Lipid profile changes have been reported since the early stages of the COVID-19 pandemic. Qin et al. reported reductions in TC and LDL-C levels in a retrospective study of 248 patients with COVID-19 [27]. Another retrospective study of 102 patients in Mexico showed that low TC and LDL-C levels were associated with more severe COVID-19 disease [28]. Consistent with those studies, TC, LDL-C, and HDL-C levels were lower, and TG levels were higher in the non-survivor group of this study.

A recent systematic review and meta-analysis compared the serum lipid levels of patients with COVID-19 vs healthy controls; severe vs non-severe COVID-19; and deceased vs surviving patients with COVID-19. Among 441 articles identified, patients with COVID-19 had lower TC and HDL-C levels compared with controls. Patients with severe COVID-19 had lower TC, LDL-C, and HDL-C levels on admission compared with patients with a non-severe disease. Deceased patients had lower TC, LDL-C, and HDL-C levels on admission compared with survivors [29]. However, studies differ in their time frame during the pandemics. Over the past two years, several developments have modulated the disease course of COVID-19. The availability of effective vaccines and the identification of several novel variants with different features added to the complexity of the COVID-19 phenotype. Therefore, studies of the association between lipid levels and COVID-19 outcomes are affected by the changes in the disease course.

Studies emanating from Turkey report that lipid levels and temporal changes in lipid profiles predict short-term mortality in patients with COVID-19 [30,31]. The future studies are needed to learn about the consequences of long-term metabolic dysregulations after COVID-19. Alterations in lipid levels can affect the onset of complex chronic diseases (e.g., cardiovascular diseases) among COVID-19 survivors.

Little is known about the biological basis for variations in HDL-C levels among individuals and populations. For instance, genome-wide association studies could not explain the differences in HDL-C [32]. Populational, geographical, ethnic, and racial differences commonly occur in HDL-C levels. The effects of COVID-19 on HDL-C levels are also highly heterogeneous [33].

Genetic epidemiology studies confirm that environmental, social, and genetic factors contribute to the Turkish population’s high prevalence of low HDL-C levels [34]. Turkish people have low levels of HDL subclass 2, apolipoprotein A-I-containing lipoproteins (LpA-I), and pre-beta-1 HDL, and increased levels of HDL3, and LpA-I/A-II particles. The Turkish Heart Study by Mahley from the Gladstone Institute and coworkers revealed that very low levels of plasma HDL-C existed in the Turkish population [34]. Mahley suggested that genetic factors partially explain the low HDL-C levels in the Turkish population [34]. Studies by Onat et al. indicate that low-grade inflammation, HDL dysfunction, and metabolic syndrome are highly prevalent in the Turkish population. Inflammation can abolish the protective effects of HDL-C [35,36]. Elevated hepatic lipase activity is clearly associated with low plasma HDL-C levels in many studies. Results of a recent genome-wide scan for plasma HDL-C in Turkish people revealed a linkage on chromosome 15q22, where the hepatic lipase gene is located, and that low HDL-C levels were 80% heritable [37]. The HDL-C levels are affected by modifiable environmental factors, particularly smoking and obesity. Cholesterol ester transfer protein *TaqIB* gene polymorphism adds to the environmental risk factors that lower plasma HDL-C levels in Turkish people [38].

It is not surprising that lipid levels change significantly in the setting of COVID-19. Cholesterol is an essential constituent of the cell membrane and signal production. Cholesterol is the precursor of steroids and hormones that are related to the stress response. Membrane cholesterol facilitates viral entry into host cells. LDL receptor-related protein-1 (LRP1) is a large multifunctional receptor that plays a role in diverse biological processes [39]. The previous studies indicated that LRP1 could be a defense mechanism against acute viral infections. The LRP1 expression is increased after *Herpesviridae* infection [40]. Eliminating LRP1 with either small interfering RNA knockdown or antibody-mediated inhibition of LRP1 increases intracellular cholesterol and eventually the infectious viral yield.

Lipid levels display important effects from the diagnosis to the treatment of viral diseases. Specific side-chain cholesterol oxidation products of the oxysterols family have been shown to inhibit a large variety of both enveloped and non-enveloped human viral pathogens. A redox imbalance can explain the individual variability of COVID-19. Coronaviruses exploit a host’s cholesterol metabolism to accommodate viral replication requirements and interfere with the host’s immune responses [41]. An *in vitro* study shows the inhibitory activity of the redox active oxysterol 27-hydroxycholesterol (27-HC) against SARS-CoV-2 without significant cytotoxicity. Serum levels of 27-HC were decreased by 50% in severe COVID-19 cases compared with the matched control group [42].

Inflammation and COVID-19 can lower the circulating levels of lipids [43]. COVID-19 is associated with a decrease in lipid levels, which serves as an indicator of the severe inflammatory burden. Inflammatory cytokine storms increase the mortality rate in COVID-19 [31]. The consequences of long-term metabolic dysregulations in the recovered patients from COVID-19 still need to be understood. Long-term effects of COVID-19 can affect the onset of complex chronic diseases (e.g., cardiovascular disease or cancer) and threaten global health in the future.

### Limitations

The limitations of the study are that it is single-centered, retrospective, and the number of patients is relatively low. The association studies between COVID-19 outcomes and HDL-C levels can display a reverse causality. Patients with low HDL-C levels have multiple comorbidities, such as vascular diseases. The patients’ HDL-C levels drop acutely during sepsis, and lower levels of HDL-C during sepsis are associated with worse clinical outcomes [12]. Before conclusions can be drawn about the association between low HDL-C levels and COVID-19 mortality, the cardiometabolic risk of patients, population characteristics, and health system dynamics in the first wave of COVID-19 studies must be assessed. Preanalytical factors can affect lipid levels [44]. Different methodologies exist for HDL-C determination (e.g., homogenized vs precipitation), and methodological differences can alter HDL-C levels and account for populational differences [45].

## CONCLUSIONS

We report that, among the lipoproteins known to have important roles in viral infections and the immune system, HDL-C predicts long-term mortality in COVID-19. This study confirms what is known and what remains to be explored in the populational differences in driving disease pathogenesis and outcome. The future horizons and high-throughput technology platforms can provide an understanding of the role of HDL-C in a host’s biology and defense against COVID-19.

## Figures and Tables

**TABLE S1:**
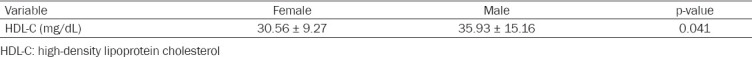
The HDL-C levels according to gender in patients with COVID-19

## References

[1] Guan WJ, Ni ZY, Hu Y, Liang WH, Ou CQ, He JX (2020). Clinical characteristics of coronavirus disease 2019 in China. N Engl J Med.

[2] Magro C, Mulvey JJ, Berlin D, Nuovo G, Salvatore S, Harp J (2020). Complement associated microvascular injury and thrombosis in the pathogenesis of severe COVID-19 infection:A report of five cases. Transl Res.

[3] Wu Z, McGoogan JM (2020). Characteristics of and important lessons from the coronavirus disease 2019 (COVID-19) outbreak in China:Summary of a report of 72 314 cases from the Chinese center for disease control and prevention. JAMA.

[4] Sardu C, Gambardella J, Morelli MB, Wang X, Marfella R, Santulli G (2020). Hypertension, thrombosis, kidney failure, and diabetes:Is COVID-19 an endothelial disease?A comprehensive evaluation of clinical and basic evidence. J Clin Med.

[5] Matarese A, Gambardella J, Sardu C, Santulli G (2020). miR-98 regulates TMPRSS2 expression in human endothelial cells:Key implications for COVID-19. Biomedicines.

[6] D'Onofrio N, Scisciola L, Sardu C, Trotta MC, De Feo M, Maiello C (2021). Glycated ACE2 receptor in diabetes:Open door for SARS-COV-2 entry in cardiomyocyte. Cardiovasc Diabetol.

[7] Sardu C, Maggi P, Messina V, Iuliano P, Sardu A, Iovinella V (2020). Could anti-hypertensive drug therapy affect the clinical prognosis of hypertensive patients with COVID-19 infection?Data from centers of Southern Italy. J Am Heart Assoc.

[8] Sardu C, D'Onofrio N, Balestrieri ML, Barbieri M, Rizzo MR, Messina V (2020). Outcomes in patients with hyperglycemia affected by COVID-19:Can we do more on glycemic control?. Diabetes Care.

[9] Sardu C, Gargiulo G, Esposito G, Paolisso G, Marfella R (2020). Impact of diabetes mellitus on clinical outcomes in patients affected by Covid-19. Cardiovasc Diabetol.

[10] Kluck GE, Yoo JA, Sakarya EH, Trigatti BL (2021). Good cholesterol gone bad?HDL and COVID-19. Int J Mol Sci.

[11] Kočar E, Režen T, Rozman D (2021). Cholesterol, lipoproteins, and COVID-19:Basic concepts and clinical applications. Biochim Biophys Acta Mol Cell Biol Lipids.

[12] Bermudes AC, De Carvalho WB, Zamberlan P, Muramoto G, Maranhão RC, Delgado AF (2018). Changes in lipid metabolism in pediatric patients with severe sepsis and septic shock. Nutrition.

[13] Wei X, Zeng W, Su J, Wan H, Yu X, Cao X (2020). Hypolipidemia is associated with the severity of COVID-19. J Clin Lipidol.

[14] Tanaka S, Couret D, Tran-Dinh A, Duranteau J, Montravers P, Schwendeman A (2020). High-density lipoproteins during sepsis:From bench to bedside. Crit Care.

[15] Mostaza JM, Salinero-Fort MA, Cardenas-Valladolid J, Rodriguez-Artalejo F, Díaz-Almiron M, Vich-Pérez P (2022). Pre-infection HDL-cholesterol levels and mortality among elderly patients infected with SARS-CoV-2. Atherosclerosis.

[16] Mahley RW, Palaoğlu KE, Atak Z, Dawson-Pepin J, Langlois AM, Cheung V (1995). Turkish heart study:Lipids, lipoproteins, and apolipoproteins. J Lipid Res.

[17] Masana L, Correig E, Ibarretxe D, Anoro E, Arroyo JA, Jericó C (2021). Low HDL and high triglycerides predict COVID-19 severity. Sci Rep.

[18] Talasaz AH, Sadeghipour P, Aghakouchakzadeh M, Dreyfus I, Kakavand H, Ariannejad H (2021). Investigating lipid-modulating agents for prevention or treatment of COVID-19:JACC state-of-the-art review. J Am Coll Cardiol.

[19] Yang R, Li X, Liu H, Zhen Y, Zhang X, Xiong Q (2020). Chest CT severity score:An imaging tool for assessing severe COVID-19. Radiol Cardiothorac Imaging.

[20] Wang Y, Hou H, Wang W, Wang W (2020). Combination of CT and RT-PCR in the screening or diagnosis of COVID-19. J Glob Health.

[21] Arnett DK, Blumenthal RS, Albert MA, Buroker AB, Goldberger ZD, Hahn EJ (2019). 2019 ACC/AHA guideline on the primary prevention of cardiovascular disease:A report of the American college of cardiology/American heart association task force on clinical practice guidelines. Circulation.

[22] Chen Y, Klein SL, Garibaldi BT, Li H, Wu C, Osevala NM (2021). Aging in COVID-19:Vulnerability, immunity and intervention. Ageing Res Rev.

[23] Tan C, Huang Y, Shi F, Tan K, Ma Q, Chen Y (2020). C-reactive protein correlates with computed tomographic findings and predicts severe COVID-19 early. J Med Virol.

[24] Gungor B, Atici A, Baycan OF, Alici G, Ozturk F, Tugrul S (2021). Elevated D-dimer levels on admission are associated with severity and increased risk of mortality in COVID-19:A systematic review and meta-analysis. Am J Emerg Med.

[25] Li J, Huang DQ, Zou B, Yang H, Hui WZ, Rui F (2021). Epidemiology of COVID-19:A systematic review and meta-analysis of clinical characteristics, risk factors, and outcomes. J Med Virol.

[26] Agouridis AP, Pagkali A, Zintzaras E, Rizos EC, Ntzani EE (2021). High-density lipoprotein cholesterol:A marker of COVID-19 infection severity?. Atheroscler Plus.

[27] Qin C, Minghan H, Ziwen Z, Yukun L (2020). Alteration of lipid profile and value of lipids in the prediction of the length of hospital stay in COVID-19 pneumonia patients. Food Sci Nutr.

[28] Osuna-Ramos JF, Rendón-Aguilar H, De Jesús-González LA, Reyes-Ruiz JM, Espinoza-Ortega AM, Ochoa-Ramírez LA (2020). Serum lipid profile changes and their clinical diagnostic significance in COVID-19 Mexican patients. MedRxiv.

[29] Chidambaram V, Geetha HS, Kumar A, Majella MG, Sivakumar RK, Voruganti D (2022). Association of lipid levels with COVID-19 Infection, disease severity and mortality:A systematic review and meta-analysis. Front Cardiovasc Med.

[30] Yıldırım ÖT, Kaya Ş (2021). The atherogenic index of plasma as a predictor of mortality in patients with COVID-19. Heart Lung.

[31] Barman HA, Pala AS, Dogan O, Atıcı A, Yumuk MT, Alici G (2021). Prognostic significance of temporal changes of lipid profile in COVID-19 patients. Obes Med.

[32] Rosenson RS, Brewer HB, Barter PJ, Björkegren JL, Chapman MJ, Gaudet D (2018). HDL and atherosclerotic cardiovascular disease:Genetic insights into complex biology. Nat Rev Cardiol.

[33] Ojo O, Wang XH, Ojo OO, Orjih E, Pavithran N, Adegboye AR (2022). The effects of COVID-19 lockdown on glycaemic control and lipid profile in patients with Type 2 diabetes:A systematic review and meta-analysis. Int J Environ Res Public Health.

[34] Mahley RW, Pépin J, Palaoğlu KE, Malloy MJ, Kane JP, Bersot TP (2000). Low levels of high density lipoproteins in Turks, a population with elevated hepatic lipase. high density lipoprotein characterization and gender-specific effects of apolipoprotein e genotype. J Lipid Res.

[35] Onat A, Can G (2014). Enhanced proinflammatory state and autoimmune activation:A breakthrough to understanding chronic diseases. Curr Pharm Des.

[36] Onat A, Hergenç G, Bulur S, Uğur M, Küçükdurmaz Z, Can G (2010). The paradox of high apolipoprotein A-I levels independently predicting incident Type-2 diabetes among Turks. Int J Cardiol.

[37] Bersot TP, Vega GL, Grundy SM, Palaoglu KE, Atagündüz P, Ozbayrakçi S (1999). Elevated hepatic lipase activity and low levels of high density lipoprotein in a normotriglyceridemic, nonobese Turkish population. J Lipid Res.

[38] Onat A, Hergenç G (2011). Low-grade inflammation, and dysfunction of high-density lipoprotein and its apolipoproteins as a major driver of cardiometabolic risk. Metabolism.

[39] He Z, Wang G, Wu J, Tang Z, Luo M (2021). The molecular mechanism of LRP1 in physiological vascular homeostasis and signal transduction pathways. Biomed Pharmacother.

[40] Gudleski-O'Regan N, Greco TM, Cristea IM, Shenk T (2012). Increased expression of LDL receptor-related protein 1 during human cytomegalovirus infection reduces virion cholesterol and infectivity. Cell Host Microbe.

[41] Dai J, Wang H, Liao Y, Tan L, Sun Y, Song C (2022). Coronavirus infection and cholesterol metabolism. Front Immunol.

[42] Marcello A, Civra A, Bonotto RM, Alves LN, Rajasekharan S, Giacobone C (2020). The cholesterol metabolite 27-hydroxycholesterol inhibits SARS-CoV-2 and is markedly decreased in COVID-19 patients. Redox Biol.

[43] Aparisi Á, Iglesias-Echeverría C, Ybarra-Falcón C, Cusácovich I, Uribarri A, García-Gómez M (2021). Low-density lipoprotein cholesterol levels are associated with poor clinical outcomes in COVID-19. Nutr Metab Cardiovasc Dis.

[44] Kouri T, Siloaho M, Pohjavaara S, Koskinen P, Malminiemi O, Pohja-Nylander P (2005). Pre-analytical factors and measurement uncertainty. Scand J Clin Lab Invest.

[45] Warnick GR, Nauck M, Rifai N (2001). Evolution of methods for measurement of HDL-cholesterol:From ultracentrifugation to homogeneous assays. Clin Chem.

